# Allied Health: engaging families in treatment

**DOI:** 10.1186/2050-2974-2-S1-P3

**Published:** 2014-11-24

**Authors:** Zarah Matthews, Heather Litchfield

**Affiliations:** 1 Royal Melbourne Hospital Eating Disorders Unit, Melbourne, Australia

## 

Family support and involvement are fundamental in supporting a patient through their recovery journey. The Eating Disorders Unit at The Royal Melbourne Hospital offers a range of family support programs in different forums as a means to provide support and education to family members. We recognize that families come in a range of forms including parents, siblings, cousins, work colleagues and friends, so providing multiple services and forums give staff the opportunity to engage numerous families. Services include the provision of information packs on admission, family / carers meetings, family therapy, information nights, Building Hope family / carers workshop and discharge meetings.

Over the past year, the allied health team working as part of The Eating Disorders Unit has significantly expanded the family work component of the program. This has been met with great feedback and results, and has made for a much more positive therapeutic relationship between patients, their families and the treating team.

